# The role of extended synaptotagmin at membrane contact sites in cancer research

**DOI:** 10.3389/fcell.2023.1291506

**Published:** 2023-11-27

**Authors:** Yuetian Pan, Dorothee Strohmer, Shikai Feng, Guibin Zhang, Hongshang Cui, Yongbin Song

**Affiliations:** ^1^ Department of General, Visceral, and Transplantation Surgery, Ludwig Maximilians University, Munich, Germany; ^2^ Department of Thoracic Surgery, Hebei General Hospital, Shijiazhuang, Hebei, China

**Keywords:** membrane contact sites, cancer progression, calcium signaling, lipid signaling, metastasis

## Abstract

Membrane contact sites (MCSs) are adjacent locations between the membranes of two different organelles and play important roles in various physiological processes, including cellular calcium and lipid signaling. In cancer research, MCSs have been proposed to regulate tumor metabolism and fate, contributing to tumor progression, and this function could be exploited for tumor therapy. However, there is little evidence on how MCSs are involved in cancer progression. In this review, we use extended synaptotagmins (E-Syts) as an entry point to describe how MCSs affect cancer progression and may be used as new diagnostic biomarkers. We then introduced the role of E-Syt and its related pathways in calcium and lipid signaling, aiming to explain how MCSs affect tumor proliferation, progression, metastasis, apoptosis, drug resistance, and treatment through calcium and lipid signaling. Generally, this review will facilitate the understanding of the complex contact biology of cancer cells.

## 1 Introduction

Cancer is the second most important cause of death worldwide and is likely to overtake ischemic heart disease as the leading cause by 2060 (Mattiuzzi and Lippi, 2019). For many tumors, effective therapy is lacking, and novel treatment approaches are urgently needed. MCSs are an underinvestigated research topic with the potential to identify new targets for anticancer therapy. Therefore, this review seeks to compile the current knowledge and explain potential future applications of MCSs.

The endoplasmic reticulum (ER) is a complex cellular network. The ER is involved in endocytosis, exocytosis and, indeed, all membrane functions via vesicle transport. However, these sites of vesicle transport exhibit only membrane contact without membrane fusion (Lee et al., 2020). These contact sites are called MCSs, and they allow the direct exchange of macromolecules. They also maintain stability between organelles and even intracellular homeostasis, making them possibly exploitable for cancer treatment (Prinz et al., 2020). However, the mechanism of MCSs in tumor metabolism is still unclear. This paper provides theoretical support for the mechanisms by which MCSs and their related proteins affect cell stability and tumor development.

**FIGURE 1:**
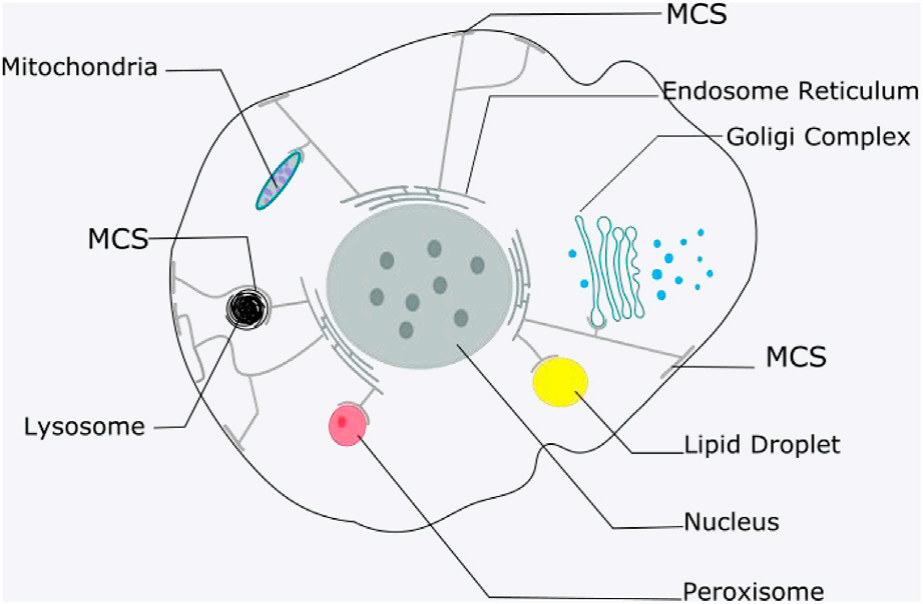
The main locations of MCSs. ER contacts with other membranous organelles and plasma membrane. It is shown that nearly all other membranous organelles, as well as the plasma membrane, have contact with the ER.

MCSs mainly encompass endoplasmic reticulum-mitochondria (ER-M), endoplasmic reticulum-plasma membrane (ER-PM), and endoplasmic reticulum-Golgi complex (ER-G) interactions. Among these contacts, ER-PM contacts have been widely studied and are ubiquitous in eukaryotic cells (Okeke et al., 2016). ER-PM contact sites were initially detected in muscle cells by electron microscopy in the 1950s (Porter and Palade, 1957). The main locations of these MCSs are shown in Figure 1.

Although ER-PM contacts have been recognized for a long time, the molecular mechanisms that drive these contacts remain unknown. Based on electron micrographs, the distance between the ER and PM at contact sites was calculated to be 10–30 nm, indicating the presence of a tether determining the unique distance and function, which corresponds to an MCS (Orci et al., 2009; Fernández-Busnadiego et al., 2015). Recently, extended synaptotagmins (E-Syts) have been shown to be active in regulating substance exchange at ER-PM contact sites (Quon and Beh, 2015; Quon et al., 2018). E-Syts are endoplasmic reticulum anchoring proteins that mediate connections between the ER and PM. They also function as a link between the two membranes, allowing substances to be transferred between them.

Synaptophysin (Syt) proteins and E-Syt proteins are related to synaptic structure and function, but they have some similarities and differences in localization and function. Syt proteins are localized mainly at the synaptic terminals of neurons and participate in the maturation and maintenance of neuronal synapses (Kochubey et al., 2016; Nazir et al., 2018). E-Syt proteins are localized mainly on the membrane of synaptic vesicles and exist as a part of the synaptic vesicle membrane (Davies et al., 2023). E-Syt is typically present in most synaptic structures, with only a few synapse types and variations that may lack or have low expression levels of E-Syt proteins (Davies et al., 2023). This difference may be related to the specific functions and characteristics of synapses. The main function of E-Syt is to maintain the structural integrity of synapses (Min et al., 2007). E-Syt proteins can also be used as synaptic markers to help researchers identify and locate synaptic structures. Overall, Syt and E-Syt play different roles in synaptic function and structure. Syt is mainly related to the process of synaptic vesicle release, while E-Syt anchors the ER to other membrane structures and is mainly related to the maintenance and labeling of synaptic structures (Ge et al., 2022).

The cytoplasmic domains of E-Syts include the synaptic mitochondrial lipid-binding protein (SMP) domain, five C2 domains in E-Syt1, and three C2 domains in E-Syt2/3 (Maeda et al., 2013). The N-terminus and C-terminus of E-Syt are both in the cytoplasm, and a transmembrane hairpin structure anchors them to the endoplasmic reticulum. The C2 domain mediates lipid-calcium binding. The N-terminal C2 domain binds calcium ions and is involved in calcium-dependent lipid binding and membrane contact. The second C2 domain does not contain calcium. The third C2 domain is important for the translocation of E-Syt proteins to the cell membrane in response to increasing cytosolic calcium levels and mediates their interactions with membranes rich in phosphatidylinositol 4,5-diphosphate [PI(4,5)P2] (Fernández-Busnadiego et al., 2015). The SMP domain is a barrel-shaped internal glycerophospholipid binding domain. It has the ability to bind to a diverse variety of lipids (Fernández-Busnadiego et al., 2015). Generally, E-Syt1 and E-Syt2 have been found to be involved in lipid transport between lipid vesicles in the cytoplasm and lipid vesicles on cell membranes, including those of the endoplasmic reticulum, Golgi apparatus, and mitochondria (Fernández-Busnadiego et al., 2015). The function of E-Syt3 is less studied, but it is also related to lipid transport (Thallmair et al., 2023).

MCSs have been identified to regulate cancer cell metabolism through a variety of activities and interactions (Prinz, 2014; Ciscato et al., 2020). In this study, E-Syt was taken as an example to explain how MCSs and their related proteins affect cell biology through calcium and lipid signaling, thus regulating tumor metabolism, and discuss the possibility of E-Syts becoming new tumor biomarkers in the future.

## 2 Functions of E-Syt in membrane contact sites

### 2.1 Calcium homeostasis (Figure 2)

ER-PM contacts mediate coupling in muscle cells and calcium entry into cells via pathways known as store-operated Ca2+ entry (SOCE) pathways (Davies et al., 2023). The SOCE pathway is an ancient and widespread calcium signaling pathway supporting calcium homeostasis that includes the STIM1 protein and Orai channels. STIM1 is a protein located on the endoplasmic reticulum membrane that can sense the status of intracellular calcium ion stores. When the calcium concentration decreases, STIM1 aggregates into clusters and moves to the region near the plasma membrane. ORAI1 is a protein located on the cell membrane that is a component of the SOCE channel and can allow extracellular calcium ions to enter the cell (Carrasco and Meyer, 2011). STIM-ORAI interactions occur at MCSs.

**FIGURE 2:**
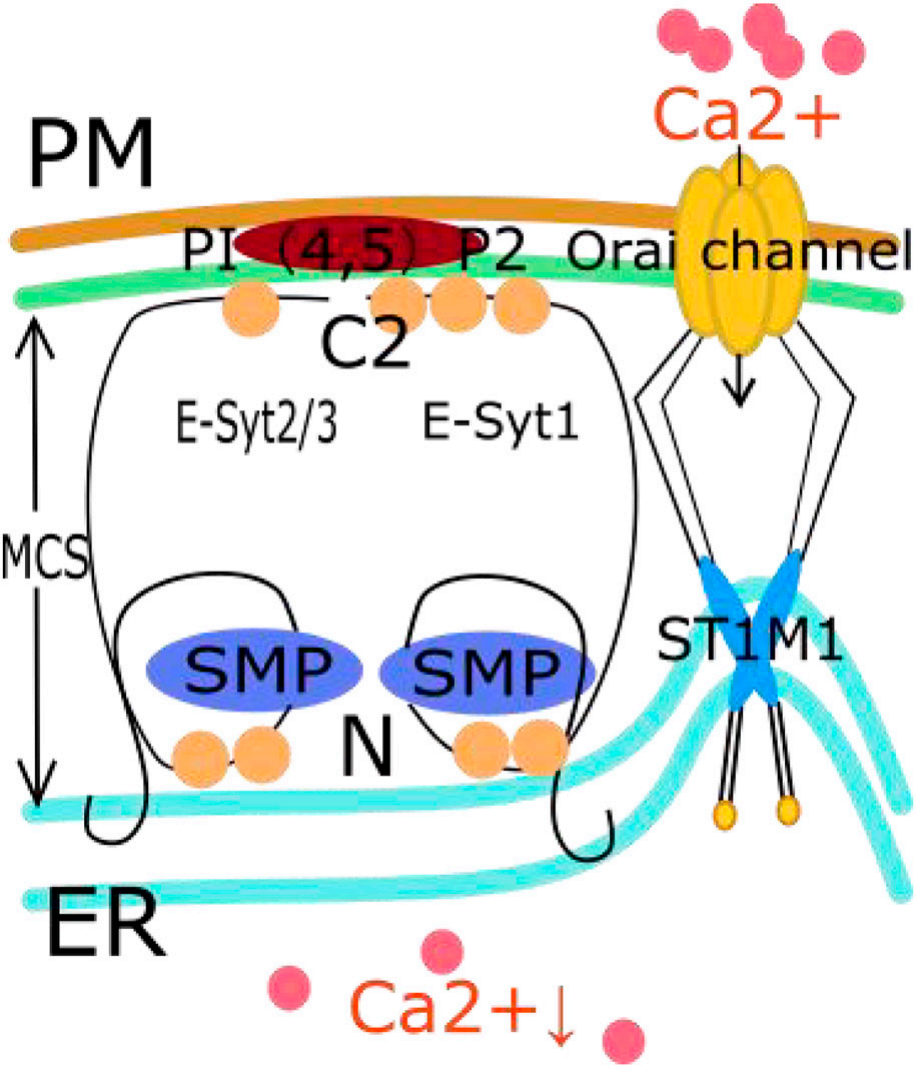
The relationship between E-Syt in MCS and SOCE pathway. First, the C2 domain of E-Syt is influenced by calcium concentration. Then E-Syt1, E-Syt2, and E-Syt3 form heterodimers. The interaction between the C2 domain of E-Syts and PI (Porter and Palade, 1957; Okeke et al., 2016) P2 on PM connects and anchors ER onto PM, forming MCS. These MCS help to narrow the distance between cell membranes such as the EER membrane, thereby promoting an increase in the local concentration of PI (Porter and Palade, 1957; Okeke et al., 2016) P2. The increased concentration of PI (Porter and Palade, 1957; Okeke et al., 2016) P2 can regulate the activation of the SOCE pathway. Then, STIM1 aggregates into clusters and moves to the area near the plasma membrane. This aggregation activates the Orai channel on the cell membrane to drive extracellular calcium influx.

In MCSs, the C2 domain of E-Syts is influenced by the calcium concentration. The C2 domain has calcium affinity and typically contains multiple calcium binding sites that attract calcium ions to interact with them (Min et al., 2007). In addition, the C2 domain helps E-Syt proteins form MCSs. During this process, the interaction between the C2 domain of an E-Syt protein and PI(4,5)P2 on the PM connects and anchors the ER to the PM (Giordano et al., 2013; Doghman-Bouguerra and Lalli, 2019). When E-Syt1, E-Syt2 and E-Syt3 form heterodimers, these heterodimers can endow PI(4,5)P2 with the ability to regulate calcium ion influx, which activates SOCE (Kang et al., 2019). During this process, E-Syt proteins can form membrane contact sites on the cell membrane as they form heterodimers. These sites help to decrease the distance between cell membranes, such as the endoplasmic reticulum membrane, and promote an increase in the local concentration of PI(4,5)P2 (Giordano et al., 2013). The increase in the concentration of PI (Porter and Palade, 1957; Okeke et al., 2016) P2 can regulate the activation of the SOCE pathway (Chang and Liou, 2016). This effect includes regulating the localization and aggregation of STIM1 by interacting with it, thereby affecting the activation of the SOCE pathway. It should be noted that the association between E-Syt proteins and the SOCE pathway is still a topic of active research, and the specific role of E-Syt3 in the SOCE pathway is still unclear. Studies have confirmed that knocking out E-Syt1 and E-Syt2 can inhibit the SOCE pathway and reduce the aggregation of STIM1 (Woo et al., 2020). However, interestingly, ER-PM connections were reduced in both HeLa and Jurkat T cells with ESYT1 and ESYT2 deletions, while SOCE was only damaged in Jurkat T cells. This indicates that the membrane binding function of E-Systs is different from its role in SOCE (Woo et al., 2020). This whole process facilitates and regulates calcium homeostasis in the ER (Kang et al., 2019). The SOCE signaling pathway is also involved in tumor progression, as we explain below. In Figure 2, we show the relationship between E-Syt in MCSs and the SOCE pathway.

### 2.2 Lipid transfer and stability

Another important role of E-Syts in MCSs is to regulate membrane lipid stability. The SMP of E-Syts is a domain involved in lipid storage and transfer. In 2020, three types of yeast endoplasmic reticulum membrane proteins were reported, namely, tricalcein-1p (Tcb1p), Tcb2p, and Tcb3p (Prinz et al., 2020). They participate in ER-PM connections and material transport and are specifically enriched in the cortical endoplasmic reticulum. These three proteins are homologs of E-Syts, with similar structural domains and N-terminal hydrophobic regions, and they are cytoplasmic synaptic proteins (Orci et al., 2009).

As stated above, the SMP of E-Syts is a structural domain involved in lipid storage and transport (Fernández-Busnadiego et al., 2015). The SMP domain is a type of lipid binding module and is often found in proteins at membrane contact sites, indicating that E-Syts can transfer lipids between the ER membrane and the PM (Reinisch and De Camilli, 2016). The SMP domain of E-Syts has a structure that is similar to that of tubular lipid binding protein (TULIP) superfamily (Schauder et al., 2014; Reinisch and De Camilli, 2016). TULIPs are also involved in lipid storage and transport. Additionally, the SMP domain is structurally similar to TULIP domains and shares common functions in lipid storage and transport (Lupas et al., 2021). Moreover, lipid transport proteins (LTPs) contain relatively small hydrophobic cavities that can accommodate a single lipid molecule (Laquitaine et al., 2006). However, the SMP dimer in E-Syts forms a hydrophobic groove that can accommodate four phospholipid molecules (Wong and Levine, 2017). These observations indicate that SMP dimers may serve as a bridge for lipid transfer. Finally, Bian et al. (2019) combined DNA origami technology with FRET-based lipid transfer to demonstrate that the SMP domain of E-Syt1 is responsible for the transfer of lipids between two membranes.

Similarly, lipids also function as second messengers in signaling pathways that control cell survival, proliferation, migration, and apoptosis. The SMP domain of E-Syts can participate in lipid storage and regulate lipid homeostasis, which also helps us to understand the ability of E-Syts to regulate the growth, metastasis, energy supply and apoptosis of tumor cells in the MCS from another perspective.

### 2.3 Coupling with other lipid transfer proteins

The lipid transport capacity of E-Syts is bilateral and driven by the lipid concentration (Orci et al., 2009). When intracellular cytoplasmic calcium levels rise, E-Syt may be engaged in lipid transport/exchange between the ER and PM. Different ER-associated lipid transfer proteins form the ER-PM interaction sites (Moser von Filseck and Drin, 2016; Tong et al., 2018; Burke, 2019). Therefore, the functions of E-Syts could be connected to those of other LTPs, such as Nir2. In fact, in ER-PM contacts, there is substantial functional coupling between E-Syts and Nir2 (Chang et al., 2013). In the absence of E-Syts, an increasing cytoplasmic calcium level causes more Nir2 to be recruited into the PM. During PI(4,5)P2 hydrolysis, Nir2 transports PA (the phosphorylated product of DAG) from the PM to the ER to recycle PA for phosphatidylinositol (PI) resynthesis in the ER (Chang and Liou, 2016; Kim et al., 2016).

Therefore, the function of E-Syts in MCSs is to connect and anchor the ER to the PM and regulate calcium homeostasis through the SOCE pathway. In addition, the SMP domain orchestrates lipid storage and transfer, which can also affect tumor growth. In addition, E-Syts and Nir2 coordinate lipid resynthesis and transport in the endoplasmic reticulum to maintain membrane lipid homeostasis.

## 3 Research on membrane contact sites in tumors

Several activities and interactions of MCSs in cancer cell metabolism have been identified and described thus far (Ciscato et al., 2020). Many MCS-related proteins, such as E-Syts, CERT, STIM1, VDAC, and Orai, have been shown to influence cancer progression and may be used as diagnostic markers (Gil-Hernández et al., 2020). The involvement of SOCE in regulating calcium signaling in various tumor progression were shown in Table 1.

**TABLE 1:** The involvement of SOCE in regulating calcium signaling in various tumor progression.

Tumor	Mechanism
Overexpression of STIM1 and Orai1 in esophageal epithelial cells and control of cell proliferation. Zhu et al. (2014)	Silencing STIM1 inhibits cell proliferation and migration, and increases apoptosis by induing G0/G1 arrest. Liu et al. (2015)
Both ORAI1 and STIM1 play a role in promoting survival and apoptosis resistance in pancreatic adenocarcinoma cell lines. SiRNA-mediated knockdown of ORAI1 and/or STIM1 increases apoptosis induced by the chemotherapy drugs 5-fluorouracil (5-FU) and gemcitabine. Singh et al. (2020)	One of the key regulators of epithelial-mesenchymal transition (EMT) is TGF-β. Previous reports have shown that it can induce EMT in breast epithelial cells. Chen et al. (2017b) Calcium channel and of calcium signaling activity in the proliferation of pancreatic cancer cells are considered to be mediated by TGF-βdownstream effectors of signal transduction. Chow et al. (2008)
In breast cancer, elevated ORAI1 expression is a feature of basal-like breast cancers, while elevated ORAI3 expression is a feature of luminal breast cancers. Azimi et al. (2019)	SOCE inhibition blocks the activation of the COX-2/PGE2 pathway. After SOCE inhibition, the proliferation and migration of breast cancer cells were inhibited. Alqinyah et al. (2023)
STIM1 and Orai1 have been shown to be upregulated in thyroid cancer patient tissue samples as well as in thyroid cancer cell lines compared with primary thyroid cells. Asghar et al. (2021)	The SOCE is decreaed in STIM1 knockdown and Orai1 knockdown thyroid cancer cells. This resulted in decrease in thyroid cancer cells proliferation. In xenograft zebrafish model, the STIM1 knockdown decreaed human thyroid tumor growth and also activated apoptosis. Asghar et al. (2021)
Overexpression of STIM1 in A549 non-small cell lung cancer (NSCLC) cells increases cell proliferation. Moreover, calcium signaling is the first step in cisplatin-induced apoptosis in NSCLC cells. Aung et al. (2009)	SOCE inhibition reduces cisplatin-dependent cell death. SOCE inhibition reduces the expression of biomarkers specific for cisplatin-induced apoptosis. Gualdani et al. (2019)

### 3.1 Calcium signaling and tumor biology

#### 3.1.1 SOCE activation participates in tumor progression

The impact of calcium signaling on tumor progression has been explored in several studies (Marchi and Pinton, 2016; Bong and Monteith, 2018). Calcium promotes the development of malignant behaviors by regulating cellular activities such as proliferation, migration, and apoptosis resistance (Morciano et al., 2018). This effect might be caused by dysregulation of pumps and channels and the resulting unequal calcium concentrations (Makena and Rao, 2020). Interestingly, several molecules associated with calcium exchange, including SOCE and calcium permeable transient receptor potential (TRP) channels, have been linked to cancer progression and metastasis (Chen et al., 2013; Yang et al., 2013).

Recently STIM1 and Orai1 have been shown to be upregulated in thyroid cancer patient tissue samples as well as in thyroid cancer cell lines compared with primary thyroid cells (Asghar et al., 2021). In addition, clinically, STIM1 has been found to be overexpressed in breast cancer, (Yang et al., 2017), colorectal cancer, (Wang et al., 2015), and lung cancer, (Zhan et al., 2015), and is associated with factors indicating poor prognosis, such as increased tumor size. Moreover, Orai1 has also been found to be overexpressed in esophageal cancer (Zhu et al., 2014; Wang et al., 2017). Then, SOCE chemical inhibitors and siRNAs targeting Orai1 and 2 have been found to inhibit calcium uptake and inhibit cell proliferation and migration (Singh et al., 2020). The SOCE is decreaed in STIM1 knockdown and Orai1 knockdown thyroid cancer cells. This resulted in decrease in thyroid cancer cells proliferation. In xenograft zebrafish model, the STIM1 knockdown decreased human thyroid tumor growth and also activated apoptosis (Asghar et al., 2021).

In conclusion, when E-Syt1 and E-Syt2/3 form heterodimers in cancer cells, these heterodimers can activate the SOCE pathway and lead to massive calcium influx. Dysregulated calcium signaling plays an important role in the uncontrolled growth and development of tumors (Tremblay and Moss, 2016). In contrast, knockdown of E-Syt1 or E-Syt2 affect the localization and aggregation of STIM1, thereby affecting the activation of the SOCE pathway (Woo et al., 2020). Figure 1 shows the involvement of SOCE in regulating calcium signaling in various tumor progression.

#### 3.1.2 Calcium shifts cancer cell metabolism toward glycolysis

**FIGURE 3:**
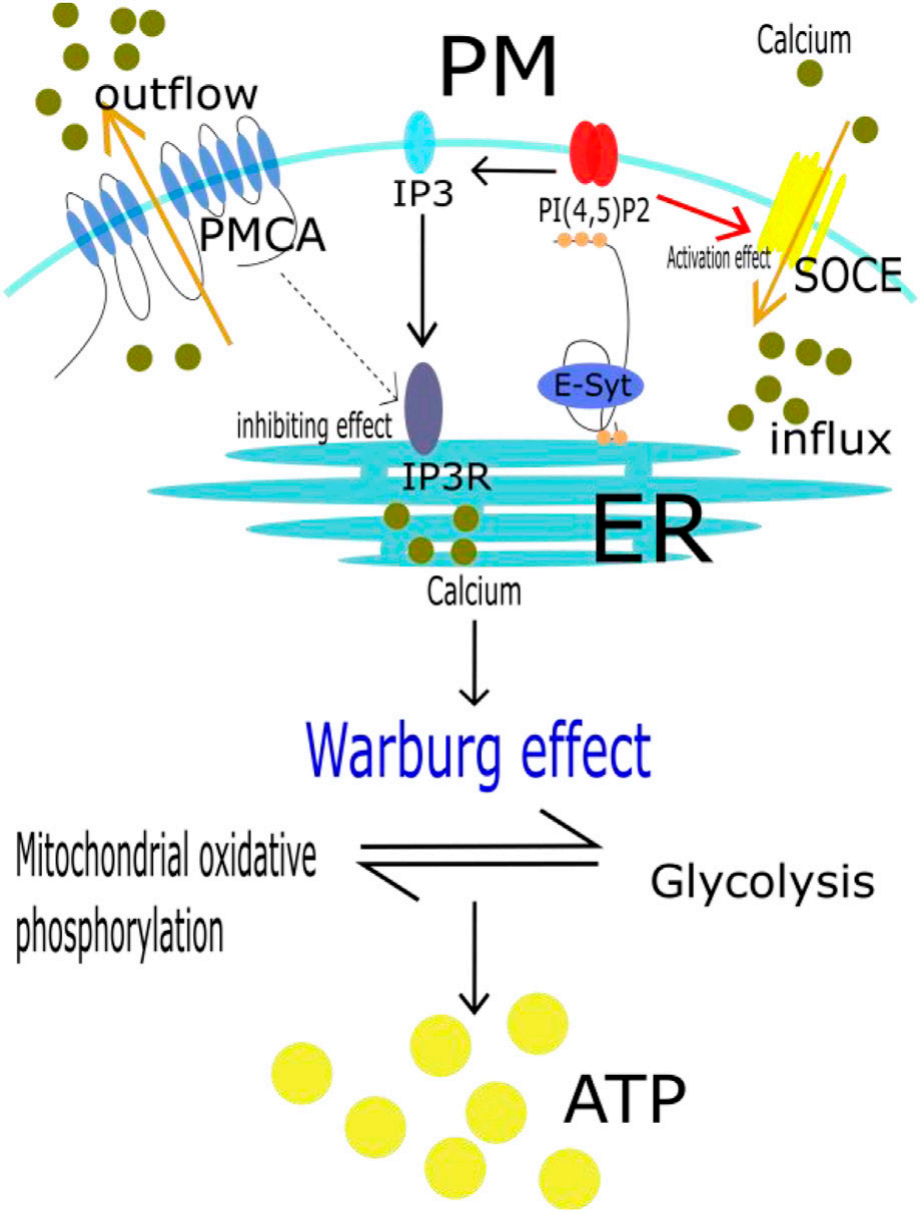
Calcium shift cancer metabolism to glycolysis. It has been shown that even in oxygen-rich conditions, tumor cells are also actived in glycolysis to produce ATP. It becauses that tumor cells have the ability, called Warburg effect, to switch between mitochondrial oxidative phosphorylation and glycolysis. In this whole process, E-Syt, MCSs, SOCE and PMCA jointly regulate the intracellular calcium concentration. High calcium concentration further regulates the Warburg effect in tumor cells, resulting in proliferation and resistance to death.

There are two pathways of glucose metabolism: mitochondrial oxidative phosphorylation and glycolysis. Glycolysis is inhibited in normal mammalian cells under aerobic conditions. However, tumor cells have the ability to switch between the two energy metabolism pathways, a phenomenon called the Warburg effect (Liberti and Locasale, 2016). Even in oxygen-rich conditions, glycolysis is activated in tumor cells, converting glucose into lactic acid to produce ATP, and this state is characterized by rapid proliferation and resistance to cell death. Glycolysis has long been considered a major metabolic process of energy production and anabolic growth in cancer cells (Porporato et al., 2018). Calcium plays an important role in this metabolic transition (Bondarenko et al., 2014; Bittremieux et al., 2016). Plasma membrane Ca2+ ATPase (PMCA) is closely involved in intracellular calcium concentration control. It reduces the calcium concentration in the cytoplasm not only by direct efflux but also by controlling inositol-1,4,5-triphosphate (IP3R) formation and reducing calcium release from the endoplasmic reticulum (Padányi et al., 2016). However, E-Syt senses a decrease in intracellular or endoplasmic reticulum calcium ion concentration in MCSs, which in turn anchors with PI (Porter and Palade, 1957; Okeke et al., 2016) P2. (Cortés et al., 2019; Dietz et al., 2022). The local concentration of PI (Porter and Palade, 1957; Okeke et al., 2016) P2 increases, and after decomposition, more IP3 is generated (Ehrlich et al., 2011; Chang and Liou, 2016). IP3 binds to receptors (IP3R) on the endoplasmic reticulum and initiates the release of calcium ions. In addition, an increase in local concentration of PI (Porter and Palade, 1957; Okeke et al., 2016) P2 can also activate the SOCE pathway and promote extracellular calcium influx (Chen YJ. et al., 2017). In other words, E-Syt, MCSs, SOCE and PMCA jointly regulate the intracellular calcium concentration. High calcium concentration further regulates the Warburg effect in tumor cells, resulting in proliferation and resistance to death (Riera Leal et al., 2020). This process is explained in Figure 3.

In recent years, cisplatin has been widely used as an anticancer drug in clinical practice that exerts effects by inhibiting the activity of key enzymes in the glycolysis pathway in tumor cells. However, overcoming cisplatin toxicity and resistance has historically been a challenge in tumor therapy. Due to the Warburg effect in tumor cells, Wang et al. (2019) designed some new platinum compounds and synthesized a triphenylphosphonium-modified terpyridine platinum (II) complex (TTP). By inhibiting thioredoxin reductase (TrxR) in mitochondria and the cytoplasm, TTP damages mitochondrial morphology and function and simultaneously inhibits mitochondrial oxidative phosphorylation and glycolysis, thus reducing the overall metabolic level in tumor cells. Finally, it can inhibit the proliferation of tumor cells and overcome cellular drug resistance.

### 3.2 Lipid signaling and tumor biology

After recognizing that calcium signaling and SOCE can regulate the proliferation and metastasis of cells, we note that lipid signaling—another major role of E-Syts in MCSs and involved in regulating the stability of membrane lipids—also plays an important role in malignant cell proliferation.

However, it is worth mentioning that although E-Syts play an important role in lipid transport and membrane lipid stability in neuronal synapses, their main function is not directly related to the biological behavior of lipids themselves (Wang et al., 2023). First, because membrane lipid molecules are components of synaptic vesicles, their transport and release are related to the activity of E-Syts (Giordano et al., 2020). Then, E-Syts participate in the perception of extracellular matrix and cellular signal transduction (He et al., 2022). In lipid droplet biology, E-synaptic proteins may affect biological behaviors related to lipid droplets by regulating intracellular signaling pathways. In addition, the interaction between Syt protein and collagen may have an impact on lipid droplet biology. These interactions may regulate the relationship between extracellular matrix tissue and lipid droplets, affecting their formation and function (Kim et al., 2009; Cohen et al., 2018). Moreover, E-Syts play important roles in the changes in lipid storage in cancer cell lipid droplets (Blücher and Stadler, 2017; Enríquez-Cortina et al., 2017; Diaz-Aragon et al., 2019).

#### 3.2.1 Uneven distribution of membrane lipids in tumors

Lipids, because of their hydrophobicity, are the physical basis of all living organisms (Nicolson, 2014). Lipids play a crucial role in cell proliferation. As cells proliferate, resynthesized lipids provide phospholipid components for proliferation and are used to form new mitotic membranes and organelle membranes. They also play important roles as signaling molecules in a variety of cellular activities (Cruz et al., 2019). However, due to the reprogramming of lipid metabolism, the newly formed membrane structures have different degrees of curvature and fluidity (Zhang et al., 2020). In normal cells, E-Syt proteins help to ensure lipid homeostasis between cell membranes and lipid vesicles. They may maintain lipid homeostasis through interactions of lipids with different cell membranes. Studies have confirmed that knockdown of E-Syt proteins can affect lipid transport functions, especially cellular processes related to lipid transport and lipid homeostasis (Saheki et al., 2016). However, studies have shown that some tumor cells may express high levels of E-Syts, leading to uneven or abnormal lipid distribution (Sun et al., 2009; Saheki et al., 2016). The main impacts of lipid structure are as follows.

First, lipid structure affects the curvature and tension of the tumor cell membrane. Phospholipids, such as phosphatidylcholine (PC), have a cylindrical shape dictated by the head-to-tail ratio and spontaneously form a bilayer in water (van Meer and de Kroon, 2011). Phosphatidic acid (PA), phosphatidylethanolamine (PE), phosphatidylserine (PS), and cholesterol (CL) are considered conical lipids because they have small heads and distorted membranes with negative curvature (Nicolson and Ash, 2014). However, lysophosphatidylcholine (LPC) has a large head-to-tail ratio, forming an inverted conical shape and resulting in positive curvature of the membrane. These lipids affect the curvature and tension of the membrane, lowering the energy needed for fusion and vesicle transport (Frohman, 2015). In addition, differences in membrane curvature and tension are involved in the processes of endocytosis/exocytosis, cell division and cell motility, which are closely related to tumor migration and invasion (Tsujita et al., 2021). For example, malignant cells have lower plasma membrane tension than normal cells, helping tumor cells offset their positive membrane curvature to facilitate their migration and invasion (Simunovic et al., 2019; Yesylevskyy et al., 2019).

The other factor is membrane fluidity. Lipid saturation is related to fluidity. Saturated lipids are beneficial for the orderly synthesis of membranes. Unsaturated lipids reduce this lipid accumulation and increase membrane fluidity (Nicolson and Ash, 2014). Dysregulation of membrane lipids results in higher or lower levels of membrane lipids in cancer cells than in normal cells, resulting in changes in membrane fluidity (Peetla et al., 2013). Cells with a low membrane lipid content have membranes that are more easily deformed and enter blood vessels more easily (Hendrich and Michalak, 2003). In addition, membrane lipids play an important role in multidrug resistance (MDR). Cancer cells have been shown to acquire MDR when the lipid components of their membranes are altered (Hendrich and Michalak, 2003). In multidrug-resistant cells, the membrane lipid content is higher, the membrane is less fluid, and the membrane permeability is lower than in normal cells, which leads to poor permeability of chemotherapy drugs in these cells (Niero et al., 2014). It has been reported that the development of nanostructured lipid carriers can overcome MDR during breast cancer (Li et al., 2018) and metastatic colon cancer (Karthika and Sureshkumar, 2019) treatment.

In summary, lipids transported through the SMP bridges of E-Syt can affect the curvature, tension, and fluidity of the membrane. Different membrane curvature and tension are involved in the processes of endocytosis/exocytosis, cell division, and cell motility, which are closely related to tumor migration and invasion (Tsujita et al., 2021). Moreover, lipid metabolic reprogramming is directly involved in the malignant transformation and progression of cells (Preta, 2020). Therefore, changes in membrane tension, fluidity and permeability in tumor cells affect the entry of chemotherapy drugs into these cells, resulting in drug resistance (Chabanel et al., 1985). Additionally, identifying the changes in lipids in the new membranes of these cells is anticipated to be greatly beneficial for the early diagnosis of cancer. Numerous human organs have been included in recent studies on the use of lipidomics in the diagnosis of cancers, including lung, (Ros-Mazurczyk et al., 2017), breast, (Corona et al., 2021), colon, (Sánchez-Martínez et al., 2015), gastric, (Wei et al., 2021), endometrial, (Seth et al., 2012), pancreatic, (Bai et al., 2021), ovarian, (Ji et al., 2020), liver, (Alannan et al., 2020), kidney (Van Hemelrijck et al., 2012) and even glial (Garanti et al., 2016) tumors. In addition, studies have shown that knockdown methods and conditions may also have an impact on lipid alterations (Woo et al., 2020). Studying the effects of E-Syt protein knockdown is crucial for obtaining a deeper understanding of the mechanisms of lipid transport, lipid homeostasis, and cell membrane function, especially in lipid-related diseases (Woo et al., 2020).

#### 3.2.2 Energy supply in tumor cells

In addition, cancer cells require large amounts of ATP to proliferate uncontrollably and invade surrounding tissues, thereby degrading the extracellular matrix (ECM) and migrating along ECM fibers (Snaebjornsson et al., 2020).

First, when lipid synthesis increases to a certain level in normal cells, lipids bind to the endoplasmic reticulum transmembrane protein INSIG, which blocks the processing of SREBP, a key transcription factor for lipid synthesis. This prevents SREBP from entering the nucleus for transcription, thus inhibiting lipid synthesis (Shao and Espenshade, 2014).

In cancer cells, however, this negative feedback system is inhibited, providing tumor cells with a steady supply of lipids (Lewis et al., 2015). Guo et al. (2019) knocked down SCAP, which inhibited the growth and adipogenic differentiation of liver cancer cells. Additionally, in prostate cancer, SREBP-2 induces the proliferation, invasion and migration of prostate cancer cells. Overexpression of SREBP-2 increases the number of prostate cancer stem cells. Genetic silencing of SREBP-2 inhibits the proliferation and metastasis of prostate cancer cells (Li et al., 2016). Through this INSIG-SREBP-SCAP pathway in MCSs, endogenous lipid synthesis is increased in tumor cells.

Second, mammalian cells can also obtain cholesterol by taking up low-density lipoprotein (LDL). Low-density lipoprotein receptors (LDLRs) are carried to the lysosome, where they are degraded by lysosomal acid lipase (LAL), resulting in the release of free cholesterol, which promotes cancer cell development. There are many adipose cells in breast cancer tissue. Coculture with cancer cells can activate lipolysis in adipose cells and release fatty acids into the extracellular space (Zhang et al., 2019). Subsequent studies have revealed that these fatty acids are in turn taken up by cancer cells, resulting in increased proliferation and migration of cancer cells (Rinninger et al., 2014).

#### 3.2.3 Lipids and tumor metastasis

For cancer cells, metastasis is difficult. Tumor cells must be detached from the primary tumor and enter blood vessels. This is a process that kills most circulating tumor cells (CTCs) (Lianidou et al., 2014). Eventually, CTCs may extravasate and find a suitable site for growth in a different tissue, where they may become resistant to most treatments until metastatic disease develops. It has been reported that lipids play an important role in this process of tumor metastasis, and this role may be related to membrane lipid saturation and transporters, but the related mechanisms still need to be elucidated in future studies. There was a study eliminated that the expression of lipid transporters were close to predict the survival rate of breast cancer (Zalba and Ten Hagen, 2017).

Jirasek et al. (2016) found a link between the aggressiveness of prostate cancer and a compound produced by cholesterol metabolism in cells. They later extended their findings to pancreatic cancer and showed that drugs used to treat atherosclerosis may have therapeutic promise in pancreatic and other cancers. The researchers found accumulation of cholesterol esters in human pancreatic cancer samples, suggesting a possible link between cholesterol esterification and metastasis (Jirasek et al., 2016). The presence of excess cholesterol results in the cholesterol storage in lipid droplets within cancer cells in the form of cholesterol esters. Inhibition of cholesterol ester formation may be a new strategy for the treatment of metastatic pancreatic cancer.

Lipids in urinary exosomes can be used as markers for prostate cancer. Recently, Skotland et al. (2017) studied the potential of lipids in urinary exosomes as biomarkers for prostate cancer. A total of 107 lipids were quantified in urinary exosomes. Several differences in lipids were discovered between urinary exosomes and exosomes derived from cell lines. The findings highlighted the importance of lipids in exosomes for biomarker research *in vivo* (Beloribi et al., 2012).

## 4 Calcium signaling in MCSs and tumor therapy

Several compounds used in chemotherapy have been shown to indirectly modulate interactions between the ER and mitochondria, which are important parts of MCSs. These drugs act on certain proteins in MCSs. For example, ML-9 (a selective myosin light chain kinase inhibitor) inhibits STIM1-plasma membrane interactions, preventing SOCE (Smyth et al., 2008). Treatment with ML-9 alone can kill prostate cancer cells. The combination of ML-9 and other anticancer drugs, such as docetaxel, can significantly promote cancer cell death (Kondratskyi et al., 2014). In addition, it is well known that specific features of the tumor microenvironment, such as hypoxia, can interfere with the function of the ER in maintaining cell homeostasis and ultimately lead to the accumulation of unfolded proteins in the ER, a condition called ER stress (Lin et al., 2019). At the same time, commonly used drugs containing metal compounds, such as cisplatin and oxaliplatin, can also modify calcium signaling and be used to treat tumors (Leo et al., 2020). If normal cells take up a large amount of calcium, ER stress and mitochondrial stress are induced, and a large amount of ROS is then produced to cause apoptosis. However, tumor cells remodel calcium signaling to avoid this increase in calcium and thus prevent the activation of this apoptotic pathway. In addition to inducing DNA damage, one of the effects of platinum drugs is to increase the influx of extracellular calcium into cells, resulting in the accumulation of large amounts of calcium in the ER and inducing ER stress (Kim and Kim, 2018). Indeed, this mechanism can also explain and be used to inhibit cisplatin-induced apoptosis and reduce cisplatin toxicity. Xu et al. (2018) demonstrated that Bcl-2 blocks cisplatin-induced apoptosis by modulating calcium signaling in various cancer cell lines. In other words, MCSs play an important role in tumor progression and metastasis, and the study of these domains provides new ideas for reducing the toxicity of antitumor drugs.

## 5 Lipid signaling and tumor therapy

Similarly, lipid signaling not only affects tumor proliferation and metabolism but also plays an important role in tumor therapy. Recent research has shown that cellular lipid metabolic reprogramming is directly involved in malignant progression and the cellular response to therapy (Liu et al., 2021).

### 5.1 Alternative lipid metabolism in immune cells can enhance the antitumor immune response

CD8^+^ T cells play a key role in antitumor immunity. A team led by Chenqi Xu and Boliang Li found that inhibition of cholesterol esterification increased the antitumor activity of CD8^+^ T cells (Yang et al., 2016). Inhibition of ACAT1 (a key cholesterol esterification enzyme) can enhance CD8^+^ T-cell proliferation. Avasimibe, a small molecule inhibitor of ACAT1, has been used to treat melanoma and has shown promising antitumor effects. In addition, intervening in lipid metabolism in immune cells can enhance anti-tumor immune response (Liu et al., 2021). These results suggest that targeting lipid metabolism in immune cells may be the key to improving antitumor immunotherapy in the future.

### 5.2 Lipids in targeted therapy

Most cancer therapies have traditionally been designed to interact with proteins and nucleic acids. However, regulation of or interactions with lipids are emerging as new therapeutic strategies. First, intervention of lipid metabolism of immune cells can enhance anti-tumor immune response. Results from Liu et al. show that reprogramming lipid metabolism can prevent senescence of effector T cells and enhance immunotherapy of melanoma and breast cancer (Liu et al., 2021). Then, reducing the level of membrane lipids by chemotherapy, radiotherapy or immunotherapy is the most advanced antitumor strategy based on lipid interactions (Farabegoli et al., 2010; Hryniewicz-Jankowska et al., 2014). Treatment with statins in combination with first-line therapy improved the therapeutic efficacy and overall survival in patients with liver cancer, acute myeloid leukemia, or refractory multiple myeloma (Mullen et al., 2016). In fact, Aberrant lipid metabolism has been recognized as a therapeutic target in liver cancer (Pope et al., 2019), pancrearic cancer (Yin et al., 2022), colorectal cancer (Chen et al., 2023) and lung cancer (Xiong et al., 2021). Combinations of different types of cell membrane lipids will be used more often as markers of prognosis and progression in the coming years, as well as being used with other biomarkers as tools for early prediction (Yan et al., 2016).

All of these observations suggest that lipid metabolism has substantial application potential for tumor diagnosis and treatment. However, there have been few investigations on the application of lipidomics in tumor therapy due to the consistent management of clinical studies and clinical observations of long-term efficacy. The introduction to MCSs and related proteins in this review provides a robust theoretical foundation for the study of lipid metabolism and regulation, considerably increasing the utility of lipidomics.

## 6 Conclusion

As the worldwide number of cancer cases increases, studies on MCSs are increasing year by year, providing the theoretical basis for the use of a large number of MCS-related proteins as new biomarkers for cancer. However, the proteins involved in MCSs and the mechanism of MCSs in tumor metabolism remain unclear. In general, we believe that one of the most attractive prospects in the field of contact science is the elucidation of the detailed mechanisms underlying the interactions between the ER and other organelles during cancer development and how they promote and/or inhibit cancer progression. In this review, we provide a wealth of information on the regulation of calcium and lipid signaling homeostasis by E-Syts and then summarize the roles of calcium and lipid signaling in tumors identified in previous research, providing a robust theoretical basis for obtaining a more complete understanding of MCS functions in the future.

## References

[B1] Alannan M. Fayyad-Kazan H. Trézéguet V. Merched A. (2020). Targeting lipid metabolism in liver cancer. Biochemistry 59 (41), 3951–3964. 10.1021/acs.biochem.0c00477 32930581

[B2] Alqinyah M. Alhamed A. S. Alnefaie H. O. Algahtani M. M. Badr A. M. Albogami A. M. (2023). Targeting store-operated calcium entry regulates the inflammation-induced proliferation and migration of breast cancer cells. Biomedicines 11 (6), 1637. 10.3390/biomedicines11061637 37371732 PMC10296208

[B3] Asghar M. Y. Lassila T. Paatero I. Nguyen V. D. Kronqvist P. Zhang J. (2021). Stromal interaction molecule 1 (STIM1) knock down attenuates invasion and proliferation and enhances the expression of thyroid-specific proteins in human follicular thyroid cancer cells. Cell Mol. Life Sci. CMLS 78 (15), 5827–5846. 10.1007/s00018-021-03880-0 34155535 PMC8316191

[B4] Aung C. S. Ye W. Plowman G. Peters A. A. Monteith G. R. Roberts-Thomson S. J. (2009). Plasma membrane calcium ATPase 4 and the remodeling of calcium homeostasis in human colon cancer cells. Carcinogenesis 30 (11), 1962–1969. 10.1093/carcin/bgp223 19755660

[B5] Azimi I. Milevskiy M. J. G. Chalmers S. B. Yapa KTDS Robitaille M. Henry C. (2019). ORAI1 and ORAI3 in breast cancer molecular subtypes and the identification of ORAI3 as a hypoxia sensitive gene and a regulator of hypoxia responses. Cancers 11 (2), 208. 10.3390/cancers11020208 30754719 PMC6406924

[B6] Bai R. Rebelo A. Kleeff J. Sunami Y. (2021). Identification of prognostic lipid droplet-associated genes in pancreatic cancer patients via bioinformatics analysis. Lipids Health Dis. 20 (1), 58. 10.1186/s12944-021-01476-y 34078402 PMC8171034

[B7] Beloribi S. Ristorcelli E. Breuzard G. Silvy F. Bertrand-Michel J. Beraud E. (2012). Exosomal lipids impact notch signaling and induce death of human pancreatic tumoral SOJ-6 cells. PloS One 7 (10), e47480. 10.1371/journal.pone.0047480 23094054 PMC3477155

[B8] Bian X. Zhang Z. Xiong Q. De Camilli P. Lin C. (2019). A programmable DNA-origami platform for studying lipid transfer between bilayers. Nat. Chem. Biol. 15 (8), 830–837. 10.1038/s41589-019-0325-3 31320758 PMC6650167

[B9] Bittremieux M. Parys J. B. Pinton P. Bultynck G. (2016). ER functions of oncogenes and tumor suppressors: modulators of intracellular Ca(2+) signaling. Biochim. Biophys. Acta 1863 (6 Pt B), 1364–1378. 10.1016/j.bbamcr.2016.01.002 26772784

[B10] Blücher C. Stadler S. C. (2017). Obesity and breast cancer: current insights on the role of fatty acids and lipid metabolism in promoting breast cancer growth and progression. Front. Endocrinol. 8, 293. 10.3389/fendo.2017.00293 PMC5670108 29163362

[B11] Bondarenko A. I. Jean-Quartier C. Parichatikanond W. Alam M. R. Waldeck-Weiermair M. Malli R. (2014). Mitochondrial Ca(2+) uniporter (MCU)-dependent and MCU-independent Ca(2+) channels coexist in the inner mitochondrial membrane. Pflugers Arch. 466 (7), 1411–1420. 10.1007/s00424-013-1383-0 24162235 PMC4020763

[B12] Bong A. H. L. Monteith G. R. (2018). Calcium signaling and the therapeutic targeting of cancer cells. Biochim. Biophys. Acta Mol. Cell Res. 1865 (11 Pt B), 1786–1794. 10.1016/j.bbamcr.2018.05.015 29842892

[B13] Burke J. E. (2019). Dynamic structural biology at the protein membrane interface. J. Biol. Chem. 294 (11), 3872–3880. 10.1074/jbc.AW118.003236 30692197 PMC6422078

[B14] Carrasco S. Meyer T. (2011). STIM proteins and the endoplasmic reticulum-plasma membrane junctions. Annu. Rev. Biochem. 80, 973–1000. 10.1146/annurev-biochem-061609-165311 21548779 PMC3897197

[B15] Chabanel A. Abbott R. E. Chien S. Schachter D. (1985). Effects of benzyl alcohol on erythrocyte shape, membrane hemileaflet fluidity and membrane viscoelasticity. Biochim. Biophys. Acta 816 (1), 142–152. 10.1016/0005-2736(85)90402-x 4005233

[B16] Chang C. L. Hsieh T. S. Yang T. T. Rothberg K. G. Azizoglu D. B. Volk E. (2013). Feedback regulation of receptor-induced Ca2+ signaling mediated by E-Syt1 and Nir2 at endoplasmic reticulum-plasma membrane junctions. Cell Rep. 5 (3), 813–825. 10.1016/j.celrep.2013.09.038 24183667

[B17] Chang C. L. Liou J. (2016). Homeostatic regulation of the PI(4,5)P2-Ca(2+) signaling system at ER-PM junctions. Biochim. Biophys. Acta 1861 (8 Pt B), 862–873. 10.1016/j.bbalip.2016.02.015 26924250 PMC4907847

[B18] Chen D. Zhou X. Yan P. Yang C. Li Y. Han L. (2023). Lipid metabolism reprogramming in colorectal cancer. J. Cell Biochem. 124 (1), 3–16. 10.1002/jcb.30347 36334309

[B19] Chen T. You Y. Jiang H. Wang Z. Z. (2017b). Epithelial-mesenchymal transition (EMT): a biological process in the development, stem cell differentiation, and tumorigenesis. J. Cell Physiol. 232 (12), 3261–3272. 10.1002/jcp.25797 28079253 PMC5507753

[B20] Chen Y. F. Chen Y. T. Chiu W. T. Shen M. R. (2013). Remodeling of calcium signaling in tumor progression. J. Biomed. Sci. 20, 23. 10.1186/1423-0127-20-23 23594099 PMC3639169

[B21] Chen Y. J. Chang C. L. Lee W. R. Liou J. (2017a). RASSF4 controls SOCE and ER-PM junctions through regulation of PI(4,5)P2. J. Cell Biol. 216 (7), 2011–2025. 10.1083/jcb.201606047 28600435 PMC5496610

[B22] Chow J. Y. C. Dong H. Quach K. T. Nguyen P. N. V. Chen K. Carethers J. M. (2008). TGF-beta mediates PTEN suppression and cell motility through calcium-dependent PKC-alpha activation in pancreatic cancer cells. Am. J. Physiol. Gastrointest. Liver Physiol. 294 (4), G899–G905. 10.1152/ajpgi.00411.2007 18239055 PMC2820122

[B23] Ciscato F. Filadi R. Masgras I. Pizzi M. Marin O. Damiano N. (2020). Hexokinase 2 displacement from mitochondria-associated membranes prompts Ca2+ -dependent death of cancer cells. EMBO Rep. 21 (7), e49117. 10.15252/embr.201949117 32383545 PMC7332982

[B24] Cohen S. Rambold A. S. Lippincott-Schwartz J. (2018). Mitochondrial and lipid droplet dynamics regulate intra- and intercellular fatty acid trafficking. Mol. Cell Oncol. 5 (5), e1043038. 10.1080/23723556.2015.1043038 30263932 PMC6154839

[B25] Corona G. Di Gregorio E. Vignoli A. Muraro E. Steffan A. Miolo G. (2021). 1H-NMR plasma lipoproteins profile analysis reveals lipid metabolism alterations in HER2-positive breast cancer patients. Cancers 13 (22), 5845. 10.3390/cancers13225845 34830999 PMC8616511

[B26] Cortés J. Hidalgo J. Aguilera S. Castro I. Brito M. Urra H. (2019). Synaptotagmin-1 overexpression under inflammatory conditions affects secretion in salivary glands from Sjögren’s syndrome patients. J. Autoimmun. 97, 88–99. 10.1016/j.jaut.2018.10.019 30391023

[B27] Cruz A. L. S. Carrossini N. Teixeira L. K. Ribeiro-Pinto L. F. Bozza P. T. Viola J. P. B. (2019). Cell cycle progression regulates biogenesis and cellular localization of lipid droplets. Mol. Cell Biol. 39 (9), 003744–e418. 10.1128/MCB.00374-18 PMC6469922 30782775

[B28] Davies C. Tulloch J. Yip E. Currie L. Colom-Cadena M. Wegmann S. (2023). Apolipoprotein E isoform does not influence trans-synaptic spread of tau pathology in a mouse model. Brain Neurosci. Adv. 7, 23982128231191046. 10.1177/23982128231191046 37600228 PMC10433884

[B29] Diaz-Aragon R. Ramirez-Ricardo J. Cortes-Reynosa P. Simoni-Nieves A. Gomez-Quiroz L. E. Perez Salazar E. (2019). Role of phospholipase D in migration and invasion induced by linoleic acid in breast cancer cells. Mol. Cell Biochem. 457 (1–2), 119–132. 10.1007/s11010-019-03517-8 30877512

[B30] Dietz J. Oelkers M. Hubrich R. Pérez-Lara A. Jahn R. Steinem C. (2022). Forces, kinetics, and fusion efficiency altered by the full-length synaptotagmin-1 -PI(4,5)P2 interaction in constrained geometries. Nano Lett. 22 (3), 1449–1455. 10.1021/acs.nanolett.1c02491 34855407

[B31] Doghman-Bouguerra M. Lalli E. (2019). ER-mitochondria interactions: both strength and weakness within cancer cells. Biochim. Biophys. Acta Mol. Cell Res. 1866 (4), 650–662. 10.1016/j.bbamcr.2019.01.009 30668969

[B32] Ehrlich L. S. Medina G. N. Carter C. A. (2011). Sprouty2 regulates PI(4,5)P2/Ca2+ signaling and HIV-1 Gag release. J. Mol. Biol. 410 (4), 716–725. 10.1016/j.jmb.2011.04.069 21762810 PMC3139110

[B33] Enríquez-Cortina C. Bello-Monroy O. Rosales-Cruz P. Souza V. Miranda R. U. Toledo-Pérez R. (2017). Cholesterol overload in the liver aggravates oxidative stress-mediated DNA damage and accelerates hepatocarcinogenesis. Oncotarget 8 (61), 104136–104148. 10.18632/oncotarget.22024 29262627 PMC5732793

[B34] Farabegoli F. Papi A. Bartolini G. Ostan R. Orlandi M. (2010). (-)-Epigallocatechin-3-gallate downregulates Pg-P and BCRP in a tamoxifen resistant MCF-7 cell line. Phytomedicine Int. J. Phytother. Phytopharm. 17 (5), 356–362. 10.1016/j.phymed.2010.01.001 20149610

[B35] Fernández-Busnadiego R. Saheki Y. De Camilli P. (2015). Three-dimensional architecture of extended synaptotagmin-mediated endoplasmic reticulum-plasma membrane contact sites. Proc. Natl. Acad. Sci. U. S. A. 112 (16), E2004–E2013. 10.1073/pnas.1503191112 25787254 PMC4413308

[B36] Frohman M. A. (2015). Role of mitochondrial lipids in guiding fission and fusion. J. Mol. Med. Berl. Ger. 93 (3), 263–269. 10.1007/s00109-014-1237-z PMC4334719 25471483

[B37] Garanti T. Stasik A. Burrow A. J. Alhnan M. A. Wan K. W. (2016). Anti-glioma activity and the mechanism of cellular uptake of asiatic acid-loaded solid lipid nanoparticles. Int. J. Pharm. 500 (1–2), 305–315. 10.1016/j.ijpharm.2016.01.018 26775062

[B38] Ge J. Bian X. Ma L. Cai Y. Li Y. Yang J. (2022). Stepwise membrane binding of extended synaptotagmins revealed by optical tweezers. Nat. Chem. Biol. 18 (3), 313–320. 10.1038/s41589-021-00914-3 34916620 PMC8891060

[B39] Gil-Hernández A. Arroyo-Campuzano M. Simoni-Nieves A. Zazueta C. Gomez-Quiroz L. E. Silva-Palacios A. (2020). Relevance of membrane contact sites in cancer progression. Front. Cell Dev. Biol. 8, 622215. 10.3389/fcell.2020.622215 33511135 PMC7835521

[B40] Giordano F. Saheki Y. Idevall-Hagren O. Colombo S. F. Pirruccello M. Milosevic I. (2013). PI(4,5)P(2)-dependent and Ca(2+)-regulated ER-PM interactions mediated by the extended synaptotagmins. Cell 153 (7), 1494–1509. 10.1016/j.cell.2013.05.026 23791178 PMC3716012

[B41] Giordano N. P. Cian M. B. Dalebroux Z. D. (2020). Outer membrane lipid secretion and the innate immune response to gram-negative bacteria. Infect. Immun. 88 (7), 009200–e1019. 10.1128/IAI.00920-19 PMC7309610 32253250

[B42] Gualdani R. de Clippele M. Ratbi I. Gailly P. Tajeddine N. (2019). Store-operated calcium entry contributes to cisplatin-induced cell death in non-small cell lung carcinoma. Cancers 11 (3), 430. 10.3390/cancers11030430 30917547 PMC6468672

[B43] Guo D. Wang Y. Wang J. Song L. Wang Z. Mao B. (2019). RA-XII suppresses the development and growth of liver cancer by inhibition of lipogenesis via SCAP-dependent SREBP supression. Mol. Basel Switz. 24 (9), E1829. 10.3390/molecules24091829 PMC6539016 31083642

[B44] He R. Li C. Liu Y. Yu H. (2022). Reconstitution and biochemical studies of extended synaptotagmin-mediated lipid transport. Methods Enzymol. 675, 33–62. 10.1016/bs.mie.2022.07.003 36220276

[B45] Hendrich A. B. Michalak K. (2003). Lipids as a target for drugs modulating multidrug resistance of cancer cells. Curr. Drug Targets 4 (1), 23–30. 10.2174/1389450033347172 12528987

[B46] Hryniewicz-Jankowska A. Augoff K. Biernatowska A. Podkalicka J. Sikorski A. F. (2014). Membrane rafts as a novel target in cancer therapy. Biochim. Biophys. Acta 1845 (2), 155–165. 10.1016/j.bbcan.2014.01.006 24480320

[B47] Ji Z. Shen Y. Feng X. Kong Y. Shao Y. Meng J. (2020). Deregulation of lipid metabolism: the critical factors in ovarian cancer. Front. Oncol. 10, 593017. 10.3389/fonc.2020.593017 33194756 PMC7604390

[B48] Jirasek A. Knief J. Deng M. Reddemann K. Thorns C. (2016). Evaluation of general and coronary atherosclerosis and malignant disease demonstrates inverse correlations for specific cancer types as well as cancer in general. Pathol. Res. Pract. 212 (11), 988–994. 10.1016/j.prp.2016.07.019 27726911

[B49] Kang F. Zhou M. Huang X. Fan J. Wei L. Boulanger J. (2019). E-syt1 Re-arranges STIM1 clusters to stabilize ring-shaped ER-PM contact sites and accelerate Ca2+ store replenishment. Sci. Rep. 9 (1), 3975. 10.1038/s41598-019-40331-0 30850711 PMC6408583

[B50] Karthika C. Sureshkumar R. (2019). Can curcumin along with chemotherapeutic drug and lipid provide an effective treatment of metastatic colon cancer and alter multidrug resistance? Med. Hypotheses 132, 109325. 10.1016/j.mehy.2019.109325 31421419

[B51] Kim C. Kim B. (2018). Anti-cancer natural products and their bioactive compounds inducing ER stress-mediated apoptosis: a review. Nutrients 10 (8), E1021. 10.3390/nu10081021 PMC6115829 30081573

[B52] Kim J. Swee M. Parks W. C. (2009). Cytosolic SYT/SS18 isoforms are actin-associated proteins that function in matrix-specific adhesion. PloS One 4 (7), e6455. 10.1371/journal.pone.0006455 19649286 PMC2714072

[B53] Kim Y. J. Guzman-Hernandez M. L. Wisniewski E. Echeverria N. Balla T. (2016). Phosphatidylinositol and phosphatidic acid transport between the ER and plasma membrane during PLC activation requires the Nir2 protein. Biochem. Soc. Trans. 44 (1), 197–201. 10.1042/BST20150187 26862206 PMC6456894

[B54] Kochubey O. Babai N. Schneggenburger R. (2016). A synaptotagmin isoform switch during the development of an identified CNS synapse. Neuron 90 (5), 984–999. 10.1016/j.neuron.2016.04.038 27210552

[B55] Kondratskyi A. Yassine M. Slomianny C. Kondratska K. Gordienko D. Dewailly E. (2014). Identification of ML-9 as a lysosomotropic agent targeting autophagy and cell death. Cell Death Dis. 5, e1193. 10.1038/cddis.2014.156 24763050 PMC4001310

[B56] Laquitaine L. Gomès E. François J. Marchive C. Pascal S. Hamdi S. (2006). Molecular basis of ergosterol-induced protection of grape against botrytis cinerea: induction of type I LTP promoter activity, WRKY, and stilbene synthase gene expression. Mol. Plant-Microbe Interact. MPMI. 19 (10), 1103–1112. 10.1094/MPMI-19-1103 17022174

[B57] Lee J. E. Cathey P. I. Wu H. Parker R. Voeltz G. K. (2020). Endoplasmic reticulum contact sites regulate the dynamics of membraneless organelles. Science 367 (6477), eaay7108. 10.1126/science.aay7108 32001628 PMC10088059

[B58] Leo M. Schmitt L. I. Küsterarent P. Kutritz A. Rassaf T. Kleinschnitz C. (2020). Platinum-based drugs cause mitochondrial dysfunction in cultured dorsal root ganglion neurons. Int. J. Mol. Sci. 21 (22), E8636. 10.3390/ijms21228636 PMC7698191 33207782

[B59] Lewis C. A. Brault C. Peck B. Bensaad K. Griffiths B. Mitter R. (2015). SREBP maintains lipid biosynthesis and viability of cancer cells under lipid- and oxygen-deprived conditions and defines a gene signature associated with poor survival in glioblastoma multiforme. Oncogene 34 (40), 5128–5140. 10.1038/onc.2014.439 25619842

[B60] Li X. Jia X. Niu H. (2018). Nanostructured lipid carriers co-delivering lapachone and doxorubicin for overcoming multidrug resistance in breast cancer therapy. Int. J. Nanomedicine 13, 4107–4119. 10.2147/IJN.S163929 30034236 PMC6047616

[B61] Li X. Wu J. B. Li Q. Shigemura K. Chung L. W. K. Huang W. C. (2016). SREBP-2 promotes stem cell-like properties and metastasis by transcriptional activation of c-Myc in prostate cancer. Oncotarget 7 (11), 12869–12884. 10.18632/oncotarget.7331 26883200 PMC4914327

[B62] Lianidou E. S. Strati A. Markou A. (2014). Circulating tumor cells as promising novel biomarkers in solid cancers. Crit. Rev. Clin. Lab. Sci. 51 (3), 160–171. 10.3109/10408363.2014.896316 24641350

[B63] Liberti M. V. Locasale J. W. (2016). The Warburg effect: how does it benefit cancer cells? Trends Biochem. Sci. 41 (3), 211–218. 10.1016/j.tibs.2015.12.001 26778478 PMC4783224

[B64] Lin Y. Jiang M. Chen W. Zhao T. Wei Y. (2019). Cancer and ER stress: mutual crosstalk between autophagy, oxidative stress and inflammatory response. Biomed. Pharmacother. Biomedecine Pharmacother. 118, 109249. 10.1016/j.biopha.2019.109249 31351428

[B65] Liu B. Yu H. H. Ye H. L. Luo Z. Y. Xiao F. (2015). Effects of stromal interacting molecule 1 gene silencing by short hairpin RNA on the biological behavior of human gastric cancer cells. Mol. Med. Rep. 12 (2), 3047–3054. 10.3892/mmr.2015.3778 25976311

[B66] Liu X. Hartman C. L. Li L. Albert C. J. Si F. Gao A. (2021). Reprogramming lipid metabolism prevents effector T cell senescence and enhances tumor immunotherapy. Sci. Transl. Med. 13 (587), eaaz6314. 10.1126/scitranslmed.aaz6314 33790024 PMC12040281

[B67] Lupas A. N. Pereira J. Alva V. Merino F. Coles M. Hartmann M. D. (2021). The breakthrough in protein structure prediction. Biochem. J. 478 (10), 1885–1890. 10.1042/BCJ20200963 34029366 PMC8166336

[B68] Maeda K. Anand K. Chiapparino A. Kumar A. Poletto M. Kaksonen M. (2013). Interactome map uncovers phosphatidylserine transport by oxysterol-binding proteins. Nature 501 (7466), 257–261. 10.1038/nature12430 23934110

[B69] Makena M. R. Rao R. (2020). Subtype specific targeting of calcium signaling in breast cancer. Cell Calcium 85, 102109. 10.1016/j.ceca.2019.102109 31783287 PMC6931135

[B70] Marchi S. Pinton P. (2016). Alterations of calcium homeostasis in cancer cells. Curr. Opin. Pharmacol. 29, 1–6. 10.1016/j.coph.2016.03.002 27043073

[B71] Mattiuzzi C. Lippi G. (2019). Current cancer epidemiology. J. Epidemiol. Glob. Health 9 (4), 217–222. 10.2991/jegh.k.191008.001 31854162 PMC7310786

[B72] Min S. W. Chang W. P. E-Syts S. T. C. (2007). E-Syts, a family of membranous Ca2+-sensor proteins with multiple C2 domains. Proc. Natl. Acad. Sci. U. S. A. 104 (10), 3823–3828. 10.1073/pnas.0611725104 17360437 PMC1820668

[B73] Morciano G. Marchi S. Morganti C. Sbano L. Bittremieux M. Kerkhofs M. (2018). Role of mitochondria-associated ER membranes in calcium regulation in cancer-specific settings. Neoplasia N. Y. N. 20 (5), 510–523. 10.1016/j.neo.2018.03.005 PMC5916088 29626751

[B74] Moser von Filseck J. Drin G. (2016). Running up that hill: how to create cellular lipid gradients by lipid counter-flows. Biochimie 130, 115–121. 10.1016/j.biochi.2016.08.001 27519300

[B75] Mullen P. J. Yu R. Longo J. Archer M. C. Penn L. Z. (2016). The interplay between cell signalling and the mevalonate pathway in cancer. Nat. Rev. Cancer 16 (11), 718–731. 10.1038/nrc.2016.76 27562463

[B76] Nazir F. H. Becker B. Brinkmalm A. Höglund K. Sandelius Å. Bergström P. (2018). Expression and secretion of synaptic proteins during stem cell differentiation to cortical neurons. Neurochem. Int. 121, 38–49. 10.1016/j.neuint.2018.10.014 30342961 PMC6232556

[B77] Nicolson G. L. (2014). The Fluid-Mosaic Model of Membrane Structure: still relevant to understanding the structure, function and dynamics of biological membranes after more than 40 years. Biochim. Biophys. Acta 1838 (6), 1451–1466. 10.1016/j.bbamem.2013.10.019 24189436

[B78] Nicolson G. L. Ash M. E. (2014). Lipid Replacement Therapy: a natural medicine approach to replacing damaged lipids in cellular membranes and organelles and restoring function. Biochim. Biophys. Acta 1838 (6), 1657–1679. 10.1016/j.bbamem.2013.11.010 24269541

[B79] Niero E. L. Rocha-Sales B. Lauand C. Cortez B. A. de Souza M. M. Rezende-Teixeira P. (2014). The multiple facets of drug resistance: one history, different approaches. J. Exp. Clin. Cancer Res. CR 33, 37. 10.1186/1756-9966-33-37 24775603 PMC4041145

[B80] Okeke E. Dingsdale H. Parker T. Voronina S. Tepikin A. V. (2016). Endoplasmic reticulum–plasma membrane junctions: structure, function and dynamics. J. Physiol. 594 (11), 2837–2847. 10.1113/JP271142 26939537 PMC4887688

[B81] Orci L. Ravazzola M. Le Coadic M. Shen W. W. Demaurex N. Cosson P. (2009). From the Cover: STIM1-induced precortical and cortical subdomains of the endoplasmic reticulum. Proc. Natl. Acad. Sci. U. S. A. 106 (46), 19358–19362. 10.1073/pnas.0911280106 19906989 PMC2775999

[B82] Padányi R. Pászty K. Hegedűs L. Varga K. Papp B. Penniston J. T. (2016). Multifaceted plasma membrane Ca(2+) pumps: from structure to intracellular Ca(2+) handling and cancer. Biochim. Biophys. Acta 1863 (6 Pt B), 1351–1363. 10.1016/j.bbamcr.2015.12.011 26707182

[B83] Peetla C. Vijayaraghavalu S. Labhasetwar V. (2013). Biophysics of cell membrane lipids in cancer drug resistance: implications for drug transport and drug delivery with nanoparticles. Adv. Drug Deliv. Rev. 65 (13–14), 1686–1698. 10.1016/j.addr.2013.09.004 24055719 PMC3840112

[B84] Pope E. D. Kimbrough E. O. Vemireddy L. P. Surapaneni P. K. Copland J. A. Mody K. (2019). Aberrant lipid metabolism as a therapeutic target in liver cancer. Expert Opin. Ther. Targets 23 (6), 473–483. 10.1080/14728222.2019.1615883 31076001 PMC6594827

[B85] Porporato P. E. Filigheddu N. Pedro JMBS Kroemer G. Galluzzi L. (2018). Mitochondrial metabolism and cancer. Cell Res. 28 (3), 265–280. 10.1038/cr.2017.155 29219147 PMC5835768

[B86] Porter K. R. Palade G. E. (1957). Studies on the endoplasmic reticulum. III. Its form and distribution in striated muscle cells. J. Biophys. Biochem. Cytol. 3 (2), 269–300. 10.1083/jcb.3.2.269 13438910 PMC2224093

[B87] Preta G. (2020). New insights into targeting membrane lipids for cancer therapy. Front. Cell Dev. Biol. 8, 571237. 10.3389/fcell.2020.571237 32984352 PMC7492565

[B88] Prinz W. A. (2014). Bridging the gap: membrane contact sites in signaling, metabolism, and organelle dynamics. J. Cell Biol. 205 (6), 759–769. 10.1083/jcb.201401126 24958771 PMC4068136

[B89] Prinz W. A. Toulmay A. Balla T. (2020). The functional universe of membrane contact sites. Nat. Rev. Mol. Cell Biol. 21 (1), 7–24. 10.1038/s41580-019-0180-9 31732717 PMC10619483

[B90] Quon E. Beh C. T. (2015). Membrane contact sites: complex zones for membrane association and lipid exchange. Lipid Insights 8 (Suppl. 1), 55–63. 10.4137/LPI.S37190 26949334 PMC4772907

[B91] Quon E. Sere Y. Y. Chauhan N. Johansen J. Sullivan D. P. Dittman J. S. (2018). Endoplasmic reticulum-plasma membrane contact sites integrate sterol and phospholipid regulation. PLoS Biol. 16 (5), e2003864. 10.1371/journal.pbio.2003864 29782498 PMC5983861

[B92] Reinisch K. M. De Camilli P. (2016). SMP-domain proteins at membrane contact sites: structure and function. Biochim. Biophys. Acta 1861 (8 Pt B), 924–927. 10.1016/j.bbalip.2015.12.003 26686281 PMC4902782

[B93] Riera Leal A. Ortiz-Lazareno P. C. Jave-Suárez L. F. Ramírez De Arellano A. Aguilar-Lemarroy A. Ortiz-García Y. M. (2020). 17β-estradiol-induced mitochondrial dysfunction and Warburg effect in cervical cancer cells allow cell survival under metabolic stress. Int. J. Oncol. 56 (1), 33–46. 10.3892/ijo.2019.4912 31746421 PMC6910176

[B94] Rinninger F. Heine M. Singaraja R. Hayden M. Brundert M. Ramakrishnan R. (2014). High density lipoprotein metabolism in low density lipoprotein receptor-deficient mice. J. Lipid Res. 55 (9), 1914–1924. 10.1194/jlr.M048819 24954421 PMC4617360

[B95] Ros-Mazurczyk M. Jelonek K. Marczyk M. Binczyk F. Pietrowska M. Polanska J. (2017). Serum lipid profile discriminates patients with early lung cancer from healthy controls. Lung Cancer Amst Neth 112, 69–74. 10.1016/j.lungcan.2017.07.036 29191603

[B96] Saheki Y. Bian X. Schauder C. M. Sawaki Y. Surma M. A. Klose C. (2016). Control of plasma membrane lipid homeostasis by the extended synaptotagmins. Nat. Cell Biol. 18 (5), 504–515. 10.1038/ncb3339 27065097 PMC4848133

[B97] Sánchez-Martínez R. Cruz-Gil S. Gómez de Cedrón M. Álvarez-Fernández M. Vargas T. Molina S. (2015). A link between lipid metabolism and epithelial-mesenchymal transition provides a target for colon cancer therapy. Oncotarget 6 (36), 38719–38736. 10.18632/oncotarget.5340 26451612 PMC4770732

[B98] Schauder C. M. Wu X. Saheki Y. Narayanaswamy P. Torta F. Wenk M. R. (2014). Structure of a lipid-bound Extended-Synaptotagmin indicates a role in lipid transfer. Nature 510 (7506), 552–555. 10.1038/nature13269 24847877 PMC4135724

[B99] Seth D. Garmo H. Wigertz A. Holmberg L. Hammar N. Jungner I. (2012). Lipid profiles and the risk of endometrial cancer in the Swedish AMORIS study. Int. J. Mol. Epidemiol. Genet. 3 (2), 122–133. 22724049 PMC3376923

[B100] Shao W. Espenshade P. J. (2014). Sterol regulatory element-binding protein (SREBP) cleavage regulates Golgi-to-endoplasmic reticulum recycling of SREBP cleavage-activating protein (SCAP). J. Biol. Chem. 289 (11), 7547–7557. 10.1074/jbc.M113.545699 24478315 PMC3953268

[B101] Simunovic M. Evergren E. Callan-Jones A. Bassereau P. (2019). Curving cells inside and out: roles of BAR domain proteins in membrane shaping and its cellular implications. Annu. Rev. Cell Dev. Biol. 35, 111–129. 10.1146/annurev-cellbio-100617-060558 31340125

[B102] Singh A. K. Roy N. K. Bordoloi D. Padmavathi G. Banik K. Khwairakpam A. D. (2020). Orai-1 and Orai-2 regulate oral cancer cell migration and colonisation by suppressing Akt/mTOR/NF-κB signalling. Life Sci. 261, 118372. 10.1016/j.lfs.2020.118372 32882268

[B103] Skotland T. Ekroos K. Kauhanen D. Simolin H. Seierstad T. Berge V. (2017). Molecular lipid species in urinary exosomes as potential prostate cancer biomarkers. Eur. J. Cancer Oxf Engl. 1990 70, 122–132. 10.1016/j.ejca.2016.10.011 27914242

[B104] Smyth J. T. Dehaven W. I. Bird G. S. Putney J. W. (2008). Ca2+-store-dependent and -independent reversal of Stim1 localization and function. J. Cell Sci. 121 (Pt 6), 762–772. 10.1242/jcs.023903 18285445 PMC2587154

[B105] Snaebjornsson M. T. Janaki-Raman S. Schulze A. (2020). Greasing the wheels of the cancer machine: the role of lipid metabolism in cancer. Cell Metab. 31 (1), 62–76. 10.1016/j.cmet.2019.11.010 31813823

[B106] Sun Y. Sun B. Wang J. Cai W. Zhao X. Zhang S. (2009). Prognostic implication of SYT-SSX fusion type and clinicopathological parameters for tumor-related death, recurrence, and metastasis in synovial sarcoma. Cancer Sci. 100 (6), 1018–1025. 10.1111/j.1349-7006.2009.01134.x 19385976 PMC11159520

[B107] Thallmair V. Schultz L. Evers S. Jolie T. Goecke C. Leitner M. G. (2023). Localization of the tubby domain, a PI(4,5)P2 biosensor, to E-Syt3-rich endoplasmic reticulum-plasma membrane junctions. J. Cell Sci. 136 (15), jcs260848. 10.1242/jcs.260848 37401342 PMC10445746

[B108] Tong J. Manik M. K. Im Y. J. (2018). Structural basis of sterol recognition and nonvesicular transport by lipid transfer proteins anchored at membrane contact sites. Proc. Natl. Acad. Sci. U. S. A. 115 (5), E856–65. 10.1073/pnas.1719709115 29339490 PMC5798383

[B109] Tremblay M. G. Moss T. (2016). Loss of all 3 Extended Synaptotagmins does not affect normal mouse development, viability or fertility. Cell Cycle Georget Tex 15 (17), 2360–2366. 10.1080/15384101.2016.1203494 PMC5004701 27399837

[B110] Tsujita K. Satow R. Asada S. Nakamura Y. Arnes L. Sako K. (2021). Homeostatic membrane tension constrains cancer cell dissemination by counteracting BAR protein assembly. Nat. Commun. 12 (1), 5930. 10.1038/s41467-021-26156-4 34635648 PMC8505629

[B111] Van Hemelrijck M. Garmo H. Hammar N. Jungner I. Walldius G. Lambe M. (2012). The interplay between lipid profiles, glucose, BMI and risk of kidney cancer in the Swedish AMORIS study. Int. J. Cancer 130 (9), 2118–2128. 10.1002/ijc.26212 21630265

[B112] van Meer G. de Kroon AIPM (2011). Lipid map of the mammalian cell. J. Cell Sci. 124 (Pt 1), 5–8. 10.1242/jcs.071233 21172818

[B113] Wang J. Y. Sun J. Huang M. Y. Wang Y. S. Hou M. F. Sun Y. (2015). STIM1 overexpression promotes colorectal cancer progression, cell motility and COX-2 expression. Oncogene 34 (33), 4358–4367. 10.1038/onc.2014.366 25381814 PMC4426254

[B114] Wang K. Zhu C. He Y. Zhang Z. Zhou W. Muhammad N. (2019). Restraining cancer cells by dual metabolic inhibition with a mitochondrion-targeted platinum(II) complex. Angew. Chem. Int. Ed. Engl. 58 (14), 4638–4643. 10.1002/anie.201900387 30693616

[B115] Wang L. Hao J. Zhang Y. Yang Z. Cao Y. Lu W. (2017). Orai1 mediates tumor-promoting store-operated Ca2+ entry in human gastrointestinal stromal tumors via c-KIT and the extracellular signal-regulated kinase pathway. Tumour Biol. J. Int. Soc. Oncodevelopmental Biol. Med. 39 (2), 1010428317691426. 10.1177/1010428317691426 28231736

[B116] Wang Y. Li Z. Wang X. Zhao Z. Jiao L. Liu R. (2023). Insights into membrane association of the SMP domain of extended synaptotagmin. Nat. Commun. 14 (1), 1504. 10.1038/s41467-023-37202-8 36932127 PMC10023780

[B117] Wei X. L. Luo T. Q. Li J. N. Xue Z. C. Wang Y. Zhang Y. (2021). Development and validation of a prognostic classifier based on lipid metabolism-related genes in gastric cancer. Front. Mol. Biosci. 8, 691143. 10.3389/fmolb.2021.691143 34277706 PMC8277939

[B118] Wong L. H. Levine T. P. (2017). Tubular lipid binding proteins (TULIPs) growing everywhere. Biochim. Biophys. Acta Mol. Cell Res. 1864 (9), 1439–1449. 10.1016/j.bbamcr.2017.05.019 28554774 PMC5507252

[B119] Woo J. S. Sun Z. Srikanth S. Gwack Y. (2020). The short isoform of extended synaptotagmin-2 controls Ca2+ dynamics in T cells via interaction with STIM1. Sci. Rep. 10 (1), 14433. 10.1038/s41598-020-71489-7 32879390 PMC7468131

[B120] Xiong R. He R. Liu B. Jiang W. Wang B. Li N. (2021). Ferroptosis: a new promising target for lung cancer therapy. Oxid. Med. Cell Longev. 2021, 8457521. 10.1155/2021/8457521 34616505 PMC8487823

[B121] Xu L. Xie Q. Qi L. Wang C. Xu N. Liu W. (2018). Bcl-2 overexpression reduces cisplatin cytotoxicity by decreasing ER-mitochondrial Ca2+ signaling in SKOV3 cells. Oncol. Rep. 39 (3), 985–992. 10.3892/or.2017.6164 29286126 PMC5802038

[B122] Yan G. Li L. Zhu B. Li Y. (2016). Lipidome in colorectal cancer. Oncotarget 7 (22), 33429–33439. 10.18632/oncotarget.7960 26967051 PMC5078107

[B123] Yang N. Tang Y. Wang F. Zhang H. Xu D. Shen Y. (2013). Blockade of store-operated Ca(2+) entry inhibits hepatocarcinoma cell migration and invasion by regulating focal adhesion turnover. Cancer Lett. 330 (2), 163–169. 10.1016/j.canlet.2012.11.040 23211538

[B124] Yang W. Bai Y. Xiong Y. Zhang J. Chen S. Zheng X. (2016). Potentiating the antitumour response of CD8(+) T cells by modulating cholesterol metabolism. Nature 531 (7596), 651–655. 10.1038/nature17412 26982734 PMC4851431

[B125] Yang Y. Jiang Z. Wang B. Chang L. Liu J. Zhang L. (2017). Expression of STIM1 is associated with tumor aggressiveness and poor prognosis in breast cancer. Pathol. Res. Pract. 213 (9), 1043–1047. 10.1016/j.prp.2017.08.006 28869106

[B126] Yesylevskyy S. Rivel T. Ramseyer C. (2019). Curvature increases permeability of the plasma membrane for ions, water and the anti-cancer drugs cisplatin and gemcitabine. Sci. Rep. 9 (1), 17214. 10.1038/s41598-019-53952-2 31748538 PMC6868207

[B127] Yin X. Xu R. Song J. Ruze R. Chen Y. Wang C. (2022). Lipid metabolism in pancreatic cancer: emerging roles and potential targets. Cancer Commun. Lond Engl. 42 (12), 1234–1256. 10.1002/cac2.12360 PMC9759769 36107801

[B128] Zalba S. Ten Hagen T. L. M. (2017). Cell membrane modulation as adjuvant in cancer therapy. Cancer Treat. Rev. 52, 48–57. 10.1016/j.ctrv.2016.10.008 27889637 PMC5195909

[B129] Zhan Z. Y. Zhong L. X. Feng M. Wang J. F. Liu D. B. Xiong J. P. (2015). Over-expression of Orai1 mediates cell proliferation and associates with poor prognosis in human non-small cell lung carcinoma. Int. J. Clin. Exp. Pathol. 8 (5), 5080–5088. 26191202 PMC4503074

[B130] Zhang Y. Li Q. Dong M. Han X. (2020). Effect of cholesterol on the fluidity of supported lipid bilayers. Colloids Surf. B Biointerfaces 196, 111353. 10.1016/j.colsurfb.2020.111353 32971441

[B131] Zhang Z. G. Zhang H. S. Sun H. L. Liu H. Y. Liu M. Y. Zhou Z. (2019). KDM5B promotes breast cancer cell proliferation and migration via AMPK-mediated lipid metabolism reprogramming. Exp. Cell Res. 379 (2), 182–190. 10.1016/j.yexcr.2019.04.006 30978340

[B132] Zhu H. Zhang H. Jin F. Fang M. Huang M. Yang C. S. (2014). Elevated Orai1 expression mediates tumor-promoting intracellular Ca2+ oscillations in human esophageal squamous cell carcinoma. Oncotarget 5 (11), 3455–3471. 10.18632/oncotarget.1903 24797725 PMC4116495

